# Disability Weight of *Clonorchis sinensis* Infection: Captured from Community Study and Model Simulation

**DOI:** 10.1371/journal.pntd.0001377

**Published:** 2011-12-13

**Authors:** Men-Bao Qian, Ying-Dan Chen, Yue-Yi Fang, Long-Qi Xu, Ting-Jun Zhu, Tan Tan, Chang-Hai Zhou, Guo-Fei Wang, Tie-Wu Jia, Guo-Jing Yang, Xiao-Nong Zhou

**Affiliations:** 1 National Institute of Parasitic Diseases, Chinese Center for Disease Control and Prevention, WHO Collaborative Center for Malaria, Schistosomiasis and Filariasis, Key Laboratory of Parasite and Vector Biology, Ministry of Health, Shanghai, People's Republic of China; 2 Center for Disease Control and Prevention of Guangdong Province, Guangzhou, People's Republic of China; 3 Center for Disease Control and Prevention of Shunde District, Shunde, People's Republic of China; 4 Jiangsu Institute of Parasitic Diseases, Wuxi, People's Republic of China; Centre Suisse de Recherches Scientifiques, United States of America

## Abstract

**Background:**

Clonorchiasis is among the most neglected tropical diseases. It is caused by ingesting raw or undercooked fish or shrimp containing the larval of *Clonorchis sinensis* and mainly endemic in Southeast Asia including China, Korea and Vietnam. The global estimations for population at risk and infected are 601 million and 35 million, respectively. However, it is still not listed among the Global Burden of Disease (GBD) and no disability weight is available for it. Disability weight reflects the average degree of loss of life value due to certain chronic disease condition and ranges between 0 (complete health) and 1 (death). It is crucial parameter for calculating the morbidity part of any disease burden in terms of disability-adjusted life years (DALYs).

**Methodology/Principal Findings:**

According to the probability and disability weight of single sequelae caused by *C. sinensis* infection, the overall disability weight could be captured through Monte Carlo simulation. The probability of single sequelae was gained from one community investigation, while the corresponding disability weight was searched from the literatures in evidence-based approach. The overall disability weights of the male and female were 0.101 and 0.050, respectively. The overall disability weights of the age group of 5–14, 15–29, 30–44, 45–59 and 60+ were 0.022, 0.052, 0.072, 0.094 and 0.118, respectively. There was some evidence showing that the disability weight and geometric mean of eggs per gram of feces (GMEPG) fitted a logarithmic equation.

**Conclusion/Significance:**

The overall disability weights of *C. sinensis* infection are differential in different sex and age groups. The disability weight captured here may be referred for estimating the disease burden of *C. sinensis* infection.

## Author Summary

Clonorchiasis is caused by eating incompletely cooked fishery product which carries the larval of *Clonorchis sinensis*. Millions of people are estimated to suffer in Southeast Asia. However, it is still among the most neglected tropical diseases due to the lack of clear evaluation, of which no disease burden available is one important reason. Our study is the first attempt to estimate the disability of *C. sinensis* infection, which reflects the average loss of life value due to some conditions and is crucial for calculating disease burden in terms of disability-adjusted life years (DALYs). After obtaining the probability and disability of single sequelae caused by *C. sinensis* infection through community investigation and literatures reviewing respectively, the overall disability was captured through model simulation. It was showed the overall disability of the male was higher than that of the female, positive correlation occurred between disability and infection intensity, and gallstone took the major attributable proportion. Thus, *C. sinensis* infection can cause apparent disability. The disability captured here may promote the further studies and benefit the final estimation of disease burden, which will promote health awareness and implementation of intervention.

## Introduction

*Clonorchis sinensis* is one of the important medical trematodiases and can cause clonorchiasis. Humans are infected through ingesting raw or undercooked fish or shrimp containing the larval (metacercariae) [1]. The adult worms live mainly in the intrahepatic bile ducts and the gallbladder, occasionally in the extrahepatic and pancreatic ducts. Most of the infected are asymptomatic, but some can develop into various symptoms and signs/complications [1], [2]. That is associated with infection intensity, duration, number of re-infection and susceptibility of the host [2]–[4]. The symptoms include fever, abdominal discomfort, weakness, anorexia, nausea, diarrhea, acute pain in the right upper quadrant and so on. Chronic infection can lead to mild changes such as inflammation, light change of liver parenchyma, thickening and expansion of ducts, ambiguity and thickening of gallbladder walls and severe sequelaes including gallstone, cholecystitis, cholangitis, hepatomegaly, cyst of liver, hypertrophy of gallbladder, polyp of gallbladder, etc [1]–[5]. The most severe clinical sequelae is cholangiocarcinoma (CCA). Owing to several studies demonstrating the relation between *C. sinensis* infection and CCA [6]–[9], it was reassessed as “carcinogenic to humans” (Group 1) by the International Agency for Research on Cancer (IARC) in 2009 [10]. Nearly 1 000 CCA cases related to *C. sinensis* infection occur annually in People's Republic of China (P.R. China), which may still be underestimated [11], [12].

Although *C. sinensis* is mainly endemic in Southeast Asia, including P.R. China, Korea and Vietnam [1], [2], [13], clonorchiasis may occur in other parts of the world where there live immigrants from endemic areas [14]–[16]. The global estimations for population at risk and infected are 601 million and 35 million, respectively [1], [17], [18]. A population of 12.5 million was estimated to be infected in P.R. China mainland in 2003, especially in southeast and northeast areas [19]. Owing to long term treatment and control efforts, other parasitic diseases such as schistosomiasis and soil transmitted helminthiases declined significantly in P.R. China [20], [21]. However, the prevalence of *C. sinensis* increased by 75% from 1990 to 2003, based on national sampling surveys [19].

Clonorchiasis is among the most neglected tropical diseases [22]. It has not been listed among the Global Burden of Disease (GBD) [18], [23]. No completely clear information yet on how severe clonorchiasis is makes it difficult to compare its impact with the morbidity of other parasitic diseases. To understand the clear epidemiological picture, WHO launched the initiative to assess the global burden of foodborne diseases including clonorchiasis in 2006 [24]. Nevertheless, up to date, it is still lack of evidence-based estimation on burden of clonorchiasis, especial the population-based data. As hoc, disability weight is still not available. Disability weight reflects the average degree of loss of life value due to certain chronic disease condition and ranges between 0 (complete health) and 1 (death) [23]. It is crucial parameter for calculating the morbidity part of any disease burden in terms of disability-adjusted life years (DALYs) that is a standard unit for health measurement in GBD. Therefore, one community-based study in P.R. China was carried out to capture the disability weight of *C. sinensis* infection through model simulation.

## Materials and Methods

### Design of the study

**Figure 1:**
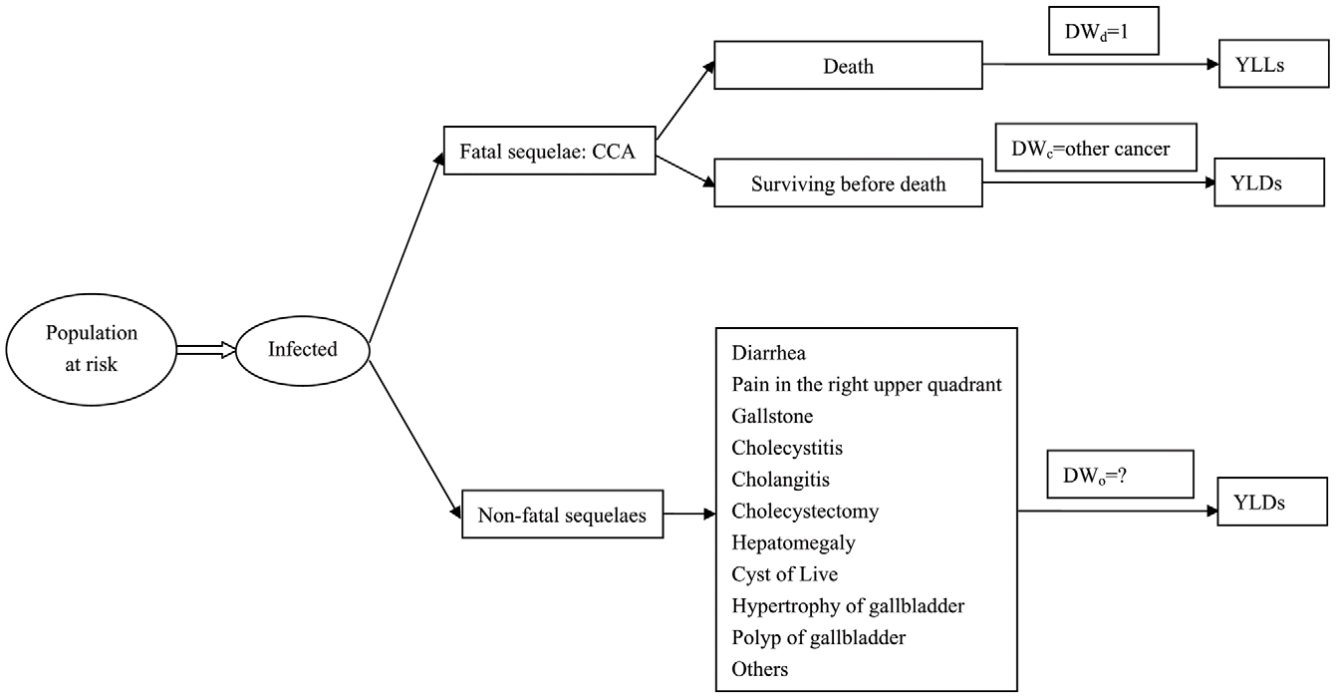
Design of the study. The disability weight of death due to CCA (DW_d_) is 1, while the disability weight of CCA patients surviving before death (DW_c_) may refer to that of other cancer. This study aims to capture the overall disability weight of *C. sinensis* infection due to non-fatal sequelaes (DWo).

DALYs as a standard unit for health measurement is consisted of premature mortality (years of life lost, or YLLs) and disability (years of life living with a disability, weighted by the severity of the disability, or YLDs) [25]. YLLs caused by *C. sinensis* infection is mainly attributable to CCA, which can be estimated separately. CCA can also contribute to YLDs, which can also be calculated separately with the disability weight referring to other cancers. Therefore, this study attempted to capture the overall disability weight of non-fatal sequelaes caused by *C. sinensis* infection (**Figure 1**). Based on the probability and corresponding disability weight of single sequelae, the overall disability weight of non-fatal sequelaes can be calculated as followings [26]:
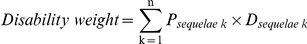
Where *P_sequelae k_* represents the probability of the k^th^ sequelae, *D_sequelae k_* represents the disability weight of the k^th^ sequelae, and n is the total number of sequelaes included. Additive effect is adopted when co-sequelaes occurs in the same person, which is implicitly indicated in the formula and the current practice of GBD.

### Study area

A village called Shibo in Shunde district, Guangdong province, P.R. China was selected as study area. The economic development of the village was relatively high and the per capita annual net income reached 11 800 RMB (about 1 815 dollars) in 2010. The selection is based on following reasons. First, Guangdong province, located in the southeast of P.R. China, has the highest prevalence of *C. sinensis* [19]. Second, Shunde district ranks among the top infection in Guangdong due to special diet habits of local people [27]. Third, the Shibo village has not yet received administration of mass drug.

### Fecal examination, questionnaire investigation and ultrasound examination

Villagers were motivated by local administrators to participate in this study. Firstly, one stool sample was collected from each voluntary participant. Triple Kato-Katz thick smears were prepared for each sample, and then microscopically examined by skilled technicians to qualify and quantify helminthes' eggs [28]. Eggs per gram of feces (EPG) was calculated by multiplying the egg number of every smear by 24 and then computing the average of three smears. Secondly, those infected with *C. sinensis* but without other helminthes were further investigated through questionnaire and ultrasound examination by trained investigators and clinical doctors, respectively. The questionnaire included 3 parts, i.e. demographical characteristics, recent health status (past one month) and past medical history. The content of health status included symptoms such as diarrhea, pain in the right upper quadrant, weakness, headache, abdominal distension, anorexia and nausea, which were used to assess sequelaes attributable to *C. sinensis* infection. The past medical history included hepatitis, diabetes, hypertension, hypotension, gastrosis and gynecological disease, which were used to exclude important confounders. The ultrasound examination involved liver and gallbladder. Any evidence of light change of liver parenchyma, thickening and expansion of ducts, ambiguity and thickening of gallbladder walls, gallstone, cholecystitis, cholangitis, cholecystectomy, hepatomegaly, cyst of liver, hypertrophy of gallbladder and polyp of gallbladder was recorded. After the investigation, the examination results were fed back to the participants. Those infected with *C. sinensis* and (or) soil transmitted helminthes (STHs) were treated with albendazole, free of charge, according to Guangdong provincial guideline for parasitic diseases control. Those with severe symptoms and (or) signs/complications were advised to search for further examination and treatment in hospital.

### Disability weight of single sequelae

The disability weights of diarrhea, low back pain/abdominal pain, gallbladder and bile duct disease, and mild/moderate hepatomeglay were available in GBD, Australia Burden of Disease (ABD) or other literatures [26], [29]–[31]. The sequelaes without corresponding disability weight, namely cyst of liver and hypertrophy of gallbladder, were assigned the same disability weight of similar sequelae, i.e mild/moderate hepatomeglay. Other symptoms such as weakness, headache, abdominal distension, anorexia and nausea were not included for analysis because of their mildness and non-specificity. Similarly, other signs such as light change of liver parenchyma, thickening and expansion of ducts, ambiguity and thickening of gallbladder walls were also excluded. In total, two symptoms and eight signs/complications were included for estimating the overall disability weight caused by *C. sinensis* infection (**Table 1**).

**Table 1:** Disability weight for single sequelae and corresponding reference.

Sequelae	Disability weights	Reference item	References
Diarrhea	0.086–0.119^a^	diarrhea	29,30
Pain in the right upper quadrant	0.060	low back pain /abdominal pain	31/26
Gallstone	0.349	gallbladder and bile duct disease	31
Cholecystitis	0.349	gallbladder and bile duct disease	31
Cholangitis	0.349	gallbladder and bile duct disease	31
cholecystectomy	0.349	gallbladder and bile duct disease	31
Hepatomegaly	0.060	mild/moderate hepatomegaly	26
Cyst of liver	0.060	mild/moderate hepatomegaly	26
Hypertrophy of gallbladder	0.060	mild/moderate hepatomegaly	26
Polyp of gallbladder	0.349	gallbladder and bile duct disease	31

a Disability weight of diarrhea varying with age group, i.e. 0.119 in 0–4, 0.094 in 5–14, 0.086 in 15–29, 0.086 in 30–44, 0.086 in 45–59, and 0.088 in 60+. An average value of 0.088 adopted for total population, the male and female.

### Probability models

To estimate the uncertainty of the results, Monte Carlo simulation was applied by WinBUGS software (http://www.mrc-bsu.cam.ac.uk/bugs/winbugs/contents.shtml). Eight subgroups were analyzed, i.e. total population, the male and female, the age group of 5–14, 15–29, 30–44, 45–59 and 60+. Following 2 000 pre-iterations, 20 000 iterations were run, when the models were all convergent. The frequency and disability weight of each sequelae were fed to the models and uniform distribution was applied. Due to the lack of priors, they were set as follows. Firstly, it is probable that all infected will fall with the two symptoms, i.e. diarrhea and pain in the right upper quadrant. Consequently, their priors were set from 0 to 1. Secondly, unlike the symptoms, the development of signs/complications was chronic and accumulated progress [32], so their priors should be narrowed. Here, they were set based on the data from the community investigation. To attain conservative results, prior of each sign/complication was set from 0 to the value which was the maximum probability of the eight signs/complications in each group. For example, in the total population, the probability of hypertrophy of gallbladder had the maximum value of 0.104 in the eight signs/complications, so priors of all eight signs/complications were set from 0 to 0.104. The same means was adopted for other groups except the age group of 5–14. In the age group of 5–14, the investigated probabilities of all eight signs/complications were 0, due to lower infection intensity and chronic progress in development of morbidity [32]. It was reasonably assumed the probabilities of above signs/complications were also approaching to 0 in this group even huger population involved. Therefore, their priors were more narrowed, set from 0 to 0.0001. The predicted probabilities, disability weights and 95% confidence interval (CI) were outputted. The WinBUGS codes used for Monte Carlo simulations were listed in **File S1**.

### Data analysis

Data were double-entered and cross-checked by EpiDate3.1 software (http://www.epidata.dk/). Analysis was run in SPSS for Windows (version11.0; SPSS Institute, Inc., Chicago, IL). The EPG was logarithmically transformed into lg(EPG), which approached to normality. Student's t test was used for comparing infection intensity in different sex. Analysis of variance (ANOVA) was employed for comparing it in different age groups, and then Dunnett test was adopted for comparison between the group of 5–14 and others. Pearson 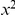 test was used to assess the association between various categories. Statistical significance was given at a *p*-value of 0.05. To attain geometric mean of EPG (GMEPG), the average of lg(EPG) was calculated and inversely logarithmically transformed.

To detect important sequelaes in the models, attributable proportion was analyzed, which denoted the proportion of disability weight of single sequelae taking in the overall one. Those contributing over 10% were considered as important ones. The relationship between disability weights and GMEPG was explored through mathematical function.

### Ethical statement

Ethical clearance had been granted by the Ethics Committee of the National Institute of Parasitic Diseases, Chinese Center for Disease Control and Prevention in Shanghai, P.R. China (Ref No: 20100525-1). The objectives, procedures and potential risks were orally explained and informed to all participants. And a written consent form was also obtained from each participant with signature of him or his proxy.

## Results

### Results of community study

**Figure 2:**
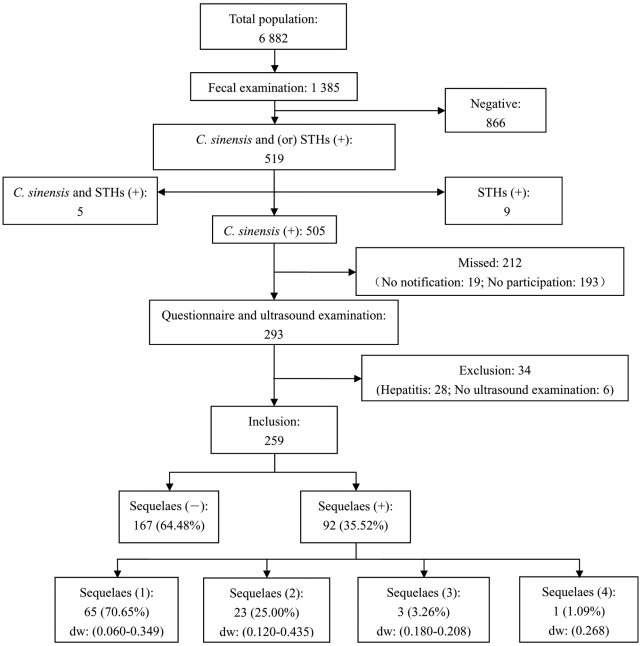
Flow chart demonstrating persons included for final analysis. 259 persons meet inclusion criteria for final analysis, of which 92 are positive for at least one of the ten sequelaes. The figures of persons with different number of sequelaes are shown and the corresponding minimum and maximum values of disability weight (dw) are also shown in brackets.

Out of 6 882 villagers, 1 385 participated in fecal examination. 519 persons were infected with *C. sinensis* and (or) STHs, and none with *Schistosoma japonicum*. Among 505 mono-infected with *C. sinensis*, 293 participated in the questionnaire investigation and ultrasound examination. Because hepatitis may result in some similar sequelaes as *C. sinensis* infection, 28 persons with hepatitis were excluded from further analysis. In addition, 6 persons without ultrasound examination result were also excluded. Therefore, 259 persons were included for final analysis, of which 167 were negative for any of the ten sequelaes. The number of persons with 1, 2, 3, and 4 sequelaes was 65, 23, 3 and 1, respectively (**Figure 2**).

There was no difference for sex composition in different age groups (*p*>0.05). The GMEPG of the male and female were 435 and 281, respectively (*p*<0.05). The GMEPG increased with the age (*p*<0.05) and it was significantly higher in age group of 30–44, 45–59 and 60+ compared to that in age group of 5–14 (*p*<0.05) (**Table 2**).

**Table 2:** The sex and age group composition of participants and corresponding GMEPG.

	age group^a^					Total	GMEPG^b^
	5–14	15–29	30–44	45–59	60+		
Male	8	13	38	56	28	143	435
Female	8	13	30	43	22	116	281
Total	16	26	68	99	50	259	358
GMEPG^c^	58	150^d^	244^e^	559^f^	702^g^	358	–

a *p* value was 0.967 for sex composition in different age groups.

b *p* value was 0.041 for GMEPG in different sex.

c *p* value was 0.000 for GMEPG in different age groups.

d *p* value was 0.137 compared to age group of 5–14.

e *p* value was 0.003 compared to age group of 5–14.

f *p* value was 0.000 compared to age group of 5–14.

g *p* value was 0.000 compared to age group of 5–14.

The frequencies and probabilities of ten sequelaes included for analysis through community investigation were listed in **Table 3**, **Table 4** **and** **Table 5**. In addition, the frequencies of weakness, headache, abdominal distension, anorexia and nausea were 20, 43, 23, 15 and 5, respectively, while that of light change of liver parenchyma, thickening and expansion of ducts, ambiguity and thickening of gallbladder walls were 53, 63 and 64, respectively. The frequencies of diabetes, hypertension, hypotension, gastrosis and gynecological disease were 4, 22, 7, 39 and 10, respectively.

**Table 3:** Probability and attributable proportion of single sequelae in total population and different sex.

Sequelae	Total						male						female					
	fre	pro	pre	dw			fre	pro	pre	dw			fre	pro	pre	dw		
				mean	95% CI	%total				mean	95% CI	%total				mean	95% CI	%total
Diarrhea	13	0.050	0.054	0.005	0.003–0.007	6	8	0.056	0.062	0.005	0.003–0.009	5	5	0.043	0.051	0.004	0.002–0.009	9
Pain in the right upper quadrant	15	0.058	0.061	0.004	0.002–0.006	5	10	0.070	0.076	0.005	0.002–0.007	4	5	0.043	0.051	0.003	0.001–0.006	6
Gallstone	19	0.073	0.074	0.026	0.017–0.035	35	11	0.077	0.082	0.028	0.015–0.044	28	8	0.069	0.054	0.019	0.011–0.024	38
Cholecystitis	1	0.004	0.008	0.003	0.000–0.007	4	0	0.000	0.007	0.002	0.000–0.009	2	1	0.009	0.017	0.006	0.001–0.016	12
Cholangitis	11	0.042	0.046	0.016	0.008–0.026	21	11	0.077	0.082	0.029	0.015–0.044	28	0	0.000	0.009	0.003	0.000–0.011	6
Cholecystectomy	1	0.004	0.008	0.003	0.000–0.007	4	1	0.007	0.014	0.005	0.001–0.014	5	0	0.000	0.008	0.003	0.000–0.011	6
Hepatomegaly	17	0.066	0.068	0.004	0.002–0.006	5	17	0.119	0.112	0.007	0.004–0.008	7	0	0.000	0.009	0.001	0.000–0.002	1
Cyst of liver	17	0.066	0.068	0.004	0.002–0.006	5	14	0.098	0.099	0.006	0.004–0.008	6	3	0.026	0.032	0.002	0.001–0.004	4
Hypertrophy of gallbladder	27	0.104	0.091	0.005	0.004–0.006	7	20	0.140	0.120	0.007	0.005–0.008	7	7	0.060	0.051	0.003	0.002–0.004	6
Polyp of gallbladder	3	0.012	0.015	0.005	0.001–0.012	7	2	0.014	0.021	0.007	0.001–0.017	7	1	0.009	0.017	0.006	0.001–0.016	12
Overall	–	–	–	0.075	0.060–0.091	100	–	–	–	0.101	0.079–0.126	100	–	–	–	0.050	0.035–0.067	100

Total: represents the total population.

fre: represents the frequency of each sequelae.

pro: represents the probability through community investigation.

pre: represents the predicted probability through model simulation.

dw: represents the predicted disability weight through model simulation.

%total: represents the attributable proportion of single sequelae taking in the overall disability weight.

**Table 4:** Probability and attributable proportion of single sequelae in different age groups (5–14, 15–29 and 30–44).

Sequelae	5–14						15–29						30–44					
	fre	pro	pre	dw			fre	pro	pre	dw			fre	pro	pre	dw		
				mean	95% CI	%total				mean	95% CI	%total				mean	95% CI	%total
Diarrhea	2	0.125	0.167	0.016	0.004–0.034	70	2	0.077	0.107	0.009	0.002–0.021	18	4	0.059	0.072	0.006	0.002–0.012	9
Pain in the right upper quadrant	1	0.063	0.111	0.007	0.001–0.017	30	1	0.038	0.072	0.004	0.001–0.011	8	4	0.059	0.071	0.004	0.001–0.008	6
Gallstone	0	0.000	0.000	0.000	0.000–0.000	0	1	0.038	0.023	0.008	0.002–0.013	16	2	0.029	0.037	0.013	0.003–0.024	18
Cholecystitis	0	0.000	0.000	0.000	0.000–0.000	0	0	0.000	0.016	0.005	0.000–0.013	11	0	0.000	0.014	0.005	0.000–0.017	7
Cholangitis	0	0.000	0.000	0.000	0.000–0.000	0	1	0.038	0.023	0.008	0.002–0.013	16	4	0.059	0.051	0.018	0.007–0.025	25
Cholecystectomy	0	0.000	0.000	0.000	0.000–0.000	0	0	0.000	0.016	0.006	0.000–0.013	11	0	0.000	0.014	0.005	0.000–0.017	7
Hepatomegaly	0	0.000	0.000	0.000	0.000–0.000	0	0	0.000	0.016	0.001	0.000–0.002	2	5	0.074	0.055	0.003	0.002–0.004	5
Cyst of liver	0	0.000	0.000	0.000	0.000–0.000	0	0	0.000	0.016	0.001	0.000–0.002	2	5	0.074	0.055	0.003	0.002–0.004	5
Hypertrophy of gallbladder	0	0.000	0.000	0.000	0.000–0.000	0	0	0.000	0.016	0.001	0.000–0.002	2	1	0.015	0.027	0.002	0.000–0.004	2
Polyp of gallbladder	0	0.000	0.000	0.000	0.000–0.000	0	1	0.038	0.023	0.008	0.002–0.013	16	2	0.029	0.037	0.013	0.003–0.025	18
Overall	–	–	–	0.022	0.008–0.043	100	–	–	–	0.052	0.033–0.071	100	–	–	–	0.072	0.049–0.097	100

fre: represents the frequency of each sequelae.

pro: represents the probability through community investigation.

pre: represents the predicted probability through model simulation.

dw: represents the predicted disability weight through model simulation.

%total: represents the attributable proportion of single sequelae taking in the overall disability weight.

**Table 5:** Probability and attributable proportion of single sequelae in different age groups (45–59 and 60+).

Sequelae	45–59						60+					
	fre	pro	pre	dw			fre	pro	pre	dw		
				mean	95% CI	%total				mean	95% CI	%total
Diarrhea	3	0.030	0.039	0.003	0.001–0.007	4	2	0.040	0.058	0.005	0.001–0.012	4
Pain in the right upper quadrant	4	0.040	0.050	0.003	0.001–0.006	3	5	0.100	0.116	0.007	0.003–0.013	6
Gallstone	10	0.101	0.096	0.034	0.019–0.045	36	6	0.120	0.133	0.047	0.020–0.080	40
Cholecystitis	1	0.010	0.020	0.007	0.001–0.019	7	0	0.000	0.019	0.007	0.000–0.025	6
Cholangitis	5	0.051	0.059	0.021	0.008–0.038	22	1	0.020	0.038	0.013	0.002–0.036	11
Cholecystectomy	1	0.010	0.020	0.007	0.001–0.019	7	0	0.000	0.019	0.007	0.000–0.024	6
Hepatomegaly	7	0.071	0.077	0.005	0.002–0.007	5	5	0.100	0.115	0.007	0.003–0.013	6
Cyst of liver	8	0.081	0.084	0.005	0.002–0.008	5	4	0.080	0.096	0.006	0.002–0.011	5
Hypertrophy of gallbladder	13	0.131	0.108	0.007	0.004–0.008	7	13	0.260	0.217	0.013	0.009–0.015	11
Polyp of gallbladder	0	0.000	0.010	0.003	0.000–0.013	4	0	0.000	0.019	0.007	0.000–0.025	6
Overall	–	–	–	0.094	0.069–0.122	100	–	–	–	0.118	0.079–0.165	100

fre: represents the frequency of each sequelae.

pro: represents the probability through community investigation.

pre: represents the predicted probability through model simulation.

dw: represents the predicted disability weight through model simulation.

%total: represents the attributable proportion of single sequelae taking in the overall disability weight.

### Outputs of model simulation

The predicted probabilities, disability weights and 95% CI were listed in **Table 3**, **Table 4** **and** **Table 5**. The overall disability weight was 0.075 (95% CI: 0.060–0.091) (**Table 3**). The overall disability weights of the male and female were 0.101 (95% CI: 0.079–0.126) and 0.050 (95% CI: 0.035–0.067), respectively (**Table 3**). The overall disability weights of the age group of 5–14, 15–29, 30–44, 45–59 and 60+ were 0.022 (95% CI: 0.008–0.043), 0.052 (95% CI: 0.033–0.071), 0.072 (95% CI: 0.049–0.097), 0.094 (95% CI: 0.069–0.122) and 0.118 (95% CI: 0.079–0.165), respectively (**Table 4** **and** **Table 5**).

### Attributable proportion

Due to the difference of probability and disability weight of each sequelae, their attributable proportion was also differential (**Table 3**, **Table 4** **and** **Table 5**). In the total population, gallstone and cholangitis took the most proportion, in sum of 56%. Gallstone and cholangitis also took the most proportion in the male, while gallstone, cholecystitis and polyp of gallbladder all took over 10% in the female. In the age group of 5–14, clinical symptoms, i.e. diarrhea and pain in the right upper quadrant, were only attributable proportion. In the age group of 15–29, diarrhea, gallstone, cholecystitis, cholangitis, cholecystectomy and polyp of gallbladder all took over 10% but less than 20%. In the age group of 30–44, cholangitis, gallstone and polyp of gallbladder ranked the top three proportion. In the age group of 45–59, gallstone and cholangitis took 36% and 22%, respectively. In the age group of 60+, gallstone, cholangitis and hypertrophy of gallbladder took 40%, 11% and 11%, respectively.

### Correlation between disability weight and infection intensity

**Figure 3:**
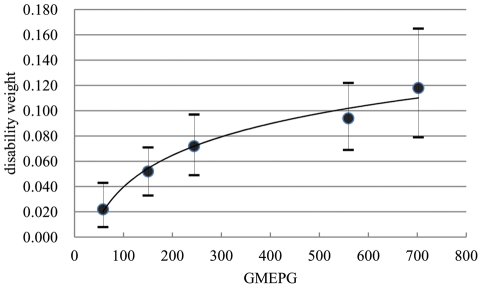
A logarithmic function between disability weight and GMEPG. The dots represent the means and error bars represent the 2.5^th^ and 97.5^th^ percentiles.

To demonstrate the relationship between disability weight and infection intensity quantitatively, mathematical function was constructed. Following the increase of GMEPG in different age groups, disability weight raised proportionally. They fitted a logarithmic equation as follows: y = 0.0362ln(x)−0.1269, where y stood for disability weight and x represented GMEPG. The correlation coefficient (R^2^) arrived at 0.9757 (*p* = 0.002) (**Figure 3**).

## Discussion

Liver flukes infection including *C. sinensis* has not been included in the GBD [18], [23] in spite of their heavy morbidity including CCA [10]. Therefore, it is the first time to present the evidence-based disability weight of liver flukes infection captured from population-based survey and model simulation. The overall disability weight of non-fatal sequelaes caused by *C. sinensis* infection is 0.075. The overall disability weights of the male and female are 0.101 and 0.050, respectively. And they are 0.022, 0.052, 0.072, 0.094 and 0.118 in the age group of 5–14, 15–29, 30–44, 45–59 and 60+, respectively. Obviously, the disability weight of the male is higher than that of the female, and the more age is, the more disability weight is. That is coherent with the difference of the GMEPG in different groups. The difference of disability weight essentially demonstrates the variance of infection intensity, in other words, the worm burden. Compared to the female, the male prefer more to eating raw fish and especially they have more chance to doing this at gathering [19], [33]. Therefore, higher exposure leads to higher infection intensity. The adult worms of *C. sinensis* can survive in human body for many years [1]. The development of major sequelaes, especially the important signs/complications, is chronic progress [32]. Furthermore, following the age's raise, the re-infection will increase. Hence, more morbidity occurs in the older. Gallstone is one of the most characteristic and frequent pathological features in clonorchiasis [5], [34]. Therefore, it takes the major attributable proportion in overall disability weight in most groups. What is an exception is in the age group of 5–14, where no clinical signs/complications take attributable proportion. That is due to their chronic clinical evolution reflecting the accumulation of worms in the body through subsequent rounds of infection [32]. The lost follow-up is quite high and not equal in different age groups with lower in the older. Those aged 45+ only took 49% in all population infected with *C. sinensis* in fecal examination, while it took 58% in the 259 persons included for final analysis. Therefore, the overall disability weight of 0.075 should not be extrapolated directly to the source population, while the age-specific disability weight will be more reasonable. Similarly, when extrapolated to other population with different age composition, age-specific disability weight should also be applied. The relationship between disability weight and infection intensity has also been explored, which fits a logarithmic equation. However, due to the lack of more points, the conclusion is not invulnerable. However, this trend deserves further study. If it occurs indeed, a mathematical model will likely become gold standard for future burden evaluation.

Various methods can be adopted for calculating disability weight. In the GBD, the person trade-off technique through expert panel was introduced, which may underestimate the disability weights of neglected tropical diseases [35]. Patients-based determination of quality of life is also valuable [36], which has been adopted in chronic schistosomiasis japonica successfully [37]. However, there is also controversy on it [38]. In a recent application for infection with STHs, no expected result was captured [39]. Thus, the operation is strict and needs high quality control. Some researchers have captured the disability weight of schistosomiasis japonica by decision-model according to the probabilities and disability weights of different morbidities [26] and the result conformed to that of patients-based quality of life assessment [37]. The new recaptured disability weight of schistosomiasis challenged existing one in GBD [40]. In this study, the similar approach was adopted.

There exists one important concern how to deal with the co-sequelaes. It is reasonable to argue that different outcomes including additive effect, synergistic effect or antagonistic effect may occur due to the difference of co-sequelaes. Especially, there exists the risk that the overall disability may be over 1 when additive effect is adopted. Consequently, to avoid this problem, the antagonistic approach was adopted in ABD [31], [41]. However, here we still adopted the additive approach. Firstly, there exists no perfect and standard way to solve this problem. Secondly, the additive approach is adopted by current practice in GBD. Thirdly, what is the most important is co-sequelaes was not serious in this study shown in **Figure 2**.

Multiparasitism is a pervasive problem, which makes the attribution of commonly encountered symptoms and signs of morbidity to particular parasites impossible [42]–[44]. For example, *schistosoma japonicum* is endemic in part of P.R. China and causes some similar sequelaes as *C. sinensis* infection, such as diarrhea, abdominal pain and hepatomegaly [26]. In addition, STHs infection can introduce similar problem. Therefore, during the design of this study, in order to avoid the confounders, the field without schistosomiasis was selected, which can be seen from the result of fecal examination. Furthermore, those infected with STHs were also excluded. Although we haven't excluded all other parasitic infections such as protozoa that can also lead to diarrhea and cyst of liver, it should not be serious problem due to the high economic development of the field with per capita annual net income over 10 000 RMB. Hepatitis is another important confounder, so persons with hepatitis were also excluded from final analysis. However, someone with other diseases such as diabetes, hypertension, hypotension, gastrosis and gynecological disease were still included. It is not reasonable and necessary to exclude them. Firstly, excluding them will diminish the sample size and lead to bias. Secondly, what is most important is that these diseases are not related to the ten sequelaes included for analysis. We have already excluded sequelaes such as weakness, headache, abdominal distension, anorexia and nausea, which could not be attributed to *C. sinensis* infection specifically.

There exist several limitations in this study. Firstly, not all sequelaes attributable to *C. sinensis* infection were included for analysis, which will lead to underestimation of the overall disability weight. Some symptoms and signs were excluded due to their mildness and non-specificity. Because the ultrasound examination only focused on liver and gallbladder, other sequelaes caused by *C. sinensis* infection such as pancreatitis [45]–[48] would be omitted. Furthermore, to exclude the potential confounders, all persons with hepatitis were excluded. However, although hepatitis is not frequently caused by *C. sinensis* infection, the potential one or more cases still exist [18]. Secondly, the disability weight of single sequelae referred to different sources. Only diarrhea is listed among the disability weights of GBD, so we have to refer to other sources such as ABD and other literatures. However, the disability weights in ABD and other literatures are not only monotonic, but also can't be discriminated for different age groups. Consequently, we have to assign the same disability weight of 0.349 to gallbladder and bile duct disease including gallstone, cholecystitis, cholangitis, cholecystectomy and polyp of gallbladder, and 0.060 to hepatomegaly, cyst of liver and hypertrophy of gallbladder for all age groups. However, we assume this is the best evidence-based. Thirdly, to elaborate which sequelaes are indeed attributable to clonorchiasis and not due to other factors, control should be introduced. But it is too difficult to search a completely matched control in field. To overcome this weakness, several potential important confounders including schistosomiasis, STHs and hepatitis were excluded in design and analysis. Although the confounding of another important factor-alcoholism has been partially avoided through exclusion of hepatitis, it may still affect the accuracy of the study, which should be solved in further study. However, the overall disability weight captured here is still not overestimated, due to incomplete inclusion of sequelaes mentioned above and conservative priors set in model simulation. Fourthly, due to the limit of sample size, a result presented as sex- and age-specific category hasn't been offered, which should also be explored in further study.

In the first WHO report on neglected tropical diseases, it is said the absence of conclusive information on the geographical distribution and burden of foodborne trematode infection including *C. sinensis* means their public–health impact may have been underestimated for decades [32]. Therefore, the disability weight captured here is expected to promote the further studies and benefit the final estimation of disease burden, which will promote health awareness and implementation of intervention.

## Supporting Information

File S1 **The WinBUGS codes of Monte Carlo simulations.**
(TXT)
